# The Impact of Sleep Disturbance on Gut Microbiota, Atrial Substrate, and Atrial Fibrillation Inducibility in Mice: A Multi-Omics Analysis

**DOI:** 10.3390/metabo12111144

**Published:** 2022-11-20

**Authors:** Kun Zuo, Chen Fang, Yuan Fu, Zheng Liu, Ye Liu, Lifeng Liu, Yuxing Wang, Hongjiang Wang, Xiandong Yin, Xiaoqing Liu, Jing Li, Jiuchang Zhong, Mulei Chen, Xinchun Yang, Li Xu

**Affiliations:** Heart Center and Beijing Key Laboratory of Hypertension, Beijing Chaoyang Hospital, Capital Medical University, Beijing 100020, China

**Keywords:** sleep deprivation, atrial fibrillation, gut microbiota, transcriptome, metabolome

## Abstract

This study examined the effect of sleep disturbance on gut microbiota (GM), atrial substrate, and atrial fibrillation (AF) inducibility. C57BL/6 mice were subjected to six weeks of sleep deprivation (SD) using the method of modified multiple-platform. Transesophageal burst pacing was performed to evaluate AF inducibility. Feces, plasma, and an atrium were collected and analyzed by 16s rRNA sequencing, liquid chromatography–mass spectrometry (LC-MS)-based metabolome, histological studies, and transcriptome. Higher AF inducibility (2/30 of control vs. 15/30 of SD, *p* = 0.001) and longer AF duration (*p* < 0.001), concomitant with aggravated fibrosis, collagen, and lipid accumulation, were seen in the SD mice compared to control mice. Meanwhile, elevated alpha diversity, higher abundance of *Flavonifractor*, *Ruminococcus,* and *Alloprevotella*, as well as imbalanced functional pathways, were observed in the gut of SD mice. Moreover, the global patterns for the plasma metabolome were altered, e.g., the decreased butanoate metabolism intermediates in SD mice. In addition, disrupted metabolic homeostasis in the SD atrium, such as fatty acid metabolism, was analyzed by the transcriptome. These results demonstrated that the crosstalk between GM and atrial metabolism might be a promising target for SD-mediated AF susceptibility.

## 1. Introduction

With the intensification of social production pressure, sleep disturbance has gradually become a common global issue. Sleep disturbance may lead to learning disabilities in children, memory impairment in people of all ages, and personality changes and depression [1,2,3]. It may also increase the risk of multiple diseases, including damage to the cardiovascular system [4,5,6]. Moreover, a recent cohort study revealed that night shift work is associated with the risk of atrial fibrillation (AF) [7]. Yet, the exact mechanism remains unclear.

Recent studies found that sleep restriction can alter the gut microbiome and affect the gut barrier integrity, which leads to systemic, low-grade inflammation [8]. Moreover, severe sleep restriction leads to the accumulation of reactive oxygen species (ROS) and consequent oxidative stress [9], while gut microbiota (GM) dysbiosis has been associated with aggravated ROS generation [10]. Inflammation and oxidative stress have been found to be involved in AF onset [11,12,13]. Moreover, previous studies have characterized the profile of dysbiotic GM in AF patients [14,15,16,17,18]. The GM contributes to host physiology by producing myriad metabolites that exert their effects within the host as signaling molecules and substrates for metabolic reactions [17,19,20,21]. For example, short-chain fatty acids (SCFA), including propionate, acetate, and butyrate, during fermentation of the dietary fiber in the colon, are responsible for the energy requirements of the colonic epithelium and its preservation by mitigating chronic inflammatory responses [22]. Notably, the existence of diurnal rhythms in a series of bacterial species and functional pathways, particularly those related to SCFA production, have been revealed by a colonic transendoscopic enteral tubing sampling of the ileocecal microbiome during the day and at night [23]. Moreover, Li et al. found that changes in GM structure and function are associated with circadian clock misalignment and sleep disturbance [24]. Hence, disrupted GM might mediate the formation of the adverse atrial matrix in AF development caused by sleep disorder.

In this study, we examined the effect of sleep disturbance on GM, atrial substrate, and AF inducibility in mice. A multi-omics approach was employed to evaluate the potential interaction among GM composition by 16s rRNA sequencing, features of GM-derived metabolites by untargeted LC-MS based metabolomics of plasma, and atrial transcriptomic profiles by transcriptome. In addition, AF inducibility was evaluated by burst pacing and histological examinations in mice following sleep deprivation (SD). We assumed that every 12 h of SD for 6 weeks might alter the GM composition, plasma metabolome, and atrial transcriptome of C57BL/6 mice, ultimately contributing to the altered atrial substrate and enhanced AF susceptibility.

## 2. Materials and Methods

### 2.1. Animal Experiment

All animal studies (including the mice euthanasia procedure) were approved by the Animal Research Ethics Committee of Capital Medical University and conducted according to the ARRIVE guidelines and the EU Directive 2010/63/EU for animal experiments.

Six- to eight-week-old male C57BL/6 mice were purchased from the Vital River Laboratory Animal Technology Company (Beijing, China). All the animals were housed in an SPF environment with a temperature of 22 ± 2 ℃, relative humidity of 55 ± 5%, and had free access to rodent chow and water. After seven days of adaption, mice were randomly assigned to the control and SD groups.

SD was performed using the modified multiple-platform method [25,26,27]. This technique effectively induces SD, as slow-wave sleep is reduced by 20%, and paradoxical sleep is eliminated [28,29]. This technique is also based on the muscle atony that accompanies paradoxical sleep [30] and produces a consistent intervention of sleep disturbance in mice [31]. The experimental groups were subjected to SD for 12 h/day (sleep window, 7:00 a.m. to 7:00 p.m.) for six consecutive weeks. Twelve narrow circular platforms (3 cm in diameter) were placed inside a tiled tank (41 × 34 × 17 cm) filled with water, 1 cm below the upper border of the platform. A total of 8 animals were placed on the platforms in each tank, following an arrangement that allowed the animals to move inside the tank and jump from one platform to the other. The animals would wake up each time they fell off the platform, which mostly occurred due to the loss of muscle tone. The blood pressure levels of mice were measured non-invasively using the tail-cuff method. The body weight of mice was recorded before and after SD.

Meanwhile, the mice in the control group remained in their home cages in the SPF room. The mouse metabolism cage was used to collect the accumulated feces over a 24 h period on days 41 and 42; samples were then stored at −80 ℃.

### 2.2. AF Inducibility by Transesophageal Burst Pacing

Transesophageal burst pacing was performed after 6 weeks of SD intervention. Mice underwent anesthesia with 1.5–2% isoflurane, and electrodes were fixed on limbs to yield a surface II-lead echocardiogram (ECG). AF was induced via transesophageal burst rapid pacing with a 1.1-Fr octapolar catheter (EPR-800, Millar Instruments, Houston, TX, USA) [32]. Each mouse was stimulated five times continuously. AF was defined as an abnormal ECG with an irregular atrial rhythm, P wave loss, and irregular R-R intervals, persisting for at least 1 s. Successful AF induction was defined as at least 2/5 episodes of AF. In addition, the percentage of mice with induced AF was displayed as AF inducibility, which was verified by an experienced cardiac electrophysiologist.

### 2.3. Histology

Using standard histological procedures, isolated atrial tissue samples were fixed in 4% paraformaldehyde and embedded in paraffin. Tissues were cut into 5 μm sections and subsequently stained with Masson’s trichrome staining and Sirius Red to evaluate left atrial (LA) fibrosis and collagen, respectively. To detect lipid deposition in the atrium, the atrial section was prepared from the frozen atrium and stained with Oil Red O staining. Micrographs were captured by a Pixera Pro600EX camera on a VANOX-S microscope (Olympus Co., Tokyo, Japan) and were analyzed using the ImageJ software (Version 1.8.0).

### 2.4. Gut Microbiota Profile of 16s rRNA

The microbiota profile of collected fecal specimens was detected using 16s rRNA gene amplicons. Firstly, total DNA was extracted through the CTAB/SDS method, and then the purity and concentration of the extracted DNA were detected by agarose gel electrophoresis. The V4 region of gut microbial 16S rRNA was characterized by the Illumina NovaSeq platform (250 bp). Quality filtering on the raw tags was performed using the fastp (Version 0.20.0) software to obtain high-quality clean tags. The clean tags were compared with the Silva database using Vsearch (Version 2.15.0) to detect the chimera sequences, and the chimera sequences were removed to obtain effective tags. Then, amplicon sequencing variants (ASVs) were obtained according to QIIME2 or DADA2 analysis process, and ASVs with an abundance < 5 were filtered out. The Silva database was utilized for taxonomic annotation.

### 2.5. Plasma Metabolomic Analyses Based on LC-MS

A plasma sample of 60 μL was placed in a 1.5 mL EP tube, mixed with 180 μL methanol, vortexed for 30 s, and ultrasonicated for 30 min at 4 °C. Samples were then kept at −20 °C for 1 h and centrifugated at 12,000 rpm at 4 °C for 15 min. Then, 100 µL supernatant was collected and mixed with 2.5 μL DL-o-chlorophenylalanine (1 mg/mL). Finally, samples were analyzed using a liquid chromatography–mass spectrometry (LC-MS) (Waters, UPLC; Thermo, Q Exactive) equipped with an ACQUITY UPLC^®^ HSS T3 (2.1 × 100 mm, 1.8 µm, Waters). The quality control (QC) sample was the equivalent mixture of all samples and was tested before, during, and after the sample injection based on LC-MS analysis. The chromatographic separation conditions included acetonitrile, 0.05% formic acid, and H_2_O as mobile phases, a column temperature of 40 °C, a flow rate of 0.3 mL/min, an injection volume of 5 μL, and an automatic injector temperature of 4 °C. The electrospray ionization (ESI) positive mode was operated using the following conditions: heater temperature was 300 °C, sheath gas flow rate of 45 arb, Aux gas flow rate of 15 arb, sweep gas flow rate of 1 arb, spray voltage of 3.0 kV, capillary temperature 350 °C, and S-Lens RF level set constant at 30%. However, in ESI negative mode, spray voltage and S-Lens RF levels were set at 3.2 kV and 60%, while other conditions remained the same as in the ESI positive mode. The metabolic data were achieved by a full scan form m/z 70-1050, and the data dependent-MS/MS (TopN = 10), with a resolution of 70,000 for MS and 17,500 for MS2, utilized high-energy collisions. All metabolomic data were prepared for feature extraction and preprocessed with Compound Discoverer 2.0 software (Thermo Fisher Scientific). Data were normalized and were then edited into a 2D data matrix by Excel 2010 software, using retention time (RT), compound molecular weight (compMW), observations (samples), and peak areas. A multivariate analysis was performed using SIMCA-P software (Umetrics AB, Umea, Sweden). Compounds were significantly distinguished between groups, identified by a variable influence on projection (VIP) > 1 and *p* < 0.05 based on the peak areas. The exact molecular mass and ms/ms value of these compounds were used to identify the metabolites related to the featured peak in the Metlin, HMDB, and KEGG databases. Using the KEGG database, we annotated and evaluated differential metabolite-related metabolic pathways.

### 2.6. Atrial Transcriptomic Signatures

The RNA extraction, sequence, and library construction were completed at Novogene Biotech Co., Ltd. (Beijing, China). Samples were sequenced by the Illumina NovaSeq 6000. The method was shown in supplementary methods. After RNA quantification and qualification, library preparation, clustering, sequencing, quality control, reads mapping to the reference genome, and quantification of gene expression level, the following analyses were carried out: differential expression analysis was performed using the DESeq2 R package (1.20.0). The resulting *p*-values were adjusted using Benjamini and Hochberg’s approach for controlling the false discovery rate. A *p*-value < 0.05 and |log2(FoldChange)| ≥ 1 were set as the threshold for significantly differential expression. Then, clusterProfiler R package (3.8.1) was used to perform Gene Set Enrichment Analysis (GSEA) of KEGG based on differential expression genes. In addition, PPI analysis of differentially expressed genes was based on the STRING database. For the species that exist in the database, we built a network of the species in the database by extracting the list of target genes from the database. Otherwise, the Diamond software (0.9.14) was used to compare the target gene sequence with the selected reference protein sequence, after which the network was established according to the known interaction of the selected reference species. Meanwhile, KEGG pathways were obtained using String (https://string-db.org/), accessed on 1 March 2022, to identify signaling pathways enriched by overlapping genes.

### 2.7. Western Blot

Atrial tissue proteins were collected after lysis with RIPA buffer containing protease and phosphatase inhibitors and centrifugation at 13,000 rpm for 15 min at 4 °C. BCA assay kit was used to quantify the protein concentration following the manufacturer’s instructions. Then, the proteins were separated using sodium dodecyl sulfate-polyacrylamide gel electrophoresis (SDS-PAGE) and blotted onto nitrocellulose membranes. Next, the membranes were blocked in 5% skim milk for 1 h and incubated overnight at 4 °C with primary antibodies against collagen I (Proteintech) and α-SMA (Proteintech). After incubation with secondary antibodies for 1 h, the membranes were detected using the Odyssey infrared imaging system (LI-COR, Lincoln, NE, USA) and analyzed by ImageJ software. GAPDH was used as an endogenous control.

### 2.8. Statistical Analysis

The Student’s *t*-test was used to compare variable differences between the control and SD groups. Pielou evenness, Shannon index, and Chao1 richness were calculated with R software (version 3.3.3, package vegan). Principal coordinate analysis (PCoA) was performed by the vegan and ape packages; all plots were visualized by the package ggplot2 in R software (version 3.3.3). Differential abundance of taxa and EC were determined using the *t*-test. Correlations among microbiota, plasma metabolites, and atrial transcriptomic features were tested using the Spearman test. All statistical tests were 2-sided, and *p* < 0.05 was considered statistically significant.

## 3. Results

### 3.1. Enhanced AF Inducibility, Atrial Lipid Accumulation, and Fibrosis in SD Mice

**Figure 1:**
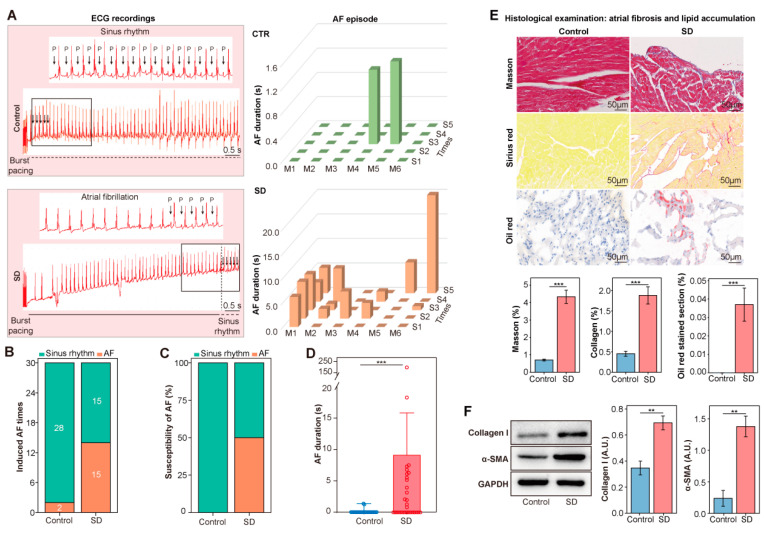
Enhanced AF inducibility, atrial lipid accumulation, and fibrosis in SD mice. (**A**) Representative image of surface ECG recordings during burst pacing in control and SD group. AF inducibility (**B**), AF susceptibility (**C**), and AF duration (**D**) in the control (*n* = 6) and SD group (*n* = 6). Chi-square test. (**E**) Representative Masson trichrome, Sirius Red, and Oil red O staining for fibrosis disarray, collagen deposition, and lipid droplets in the atrium. (**F**) Representative Western blot and the expression levels of collagen I and α-SMA in mouse atria of the two groups. Data are presented as the mean ± SEM. **, *p* < 0.01;***, *p* < 0.001; *n* = 3 for each group, Student’s *t*-test.

In order to determine whether SD intervention affects susceptibility to AF, a burst pacing test was performed in SD (*n* = 6) and control (*n* = 6) mice. Compared to control mice, SD mice were more susceptible to atrial arrhythmia (AF was seen 3/6 (50.00%) in SD mice vs. 0/6 (0.00%) in the control mice; *p* = 0.128) (Figure 1A–D). Meanwhile, there were no significant differences in systolic (*p* = 0.251) or diastolic (*p* = 0.178) blood pressure (Figure S1B) and six-week weight gain (*p* = 0.085, Figure S1A) between the two groups.

Moreover, Masson’s trichrome staining showed that atrial fibrosis was evidently increased in SD mice (*p* < 0.001), and the collagen content assessed by Sirius Red exhibited a similar tendency (*p* < 0.001), which was accompanied by the increased expression of collagen I and α-SMA (Figure 1F). Notably, Oil Red O staining of atrial sections demonstrated significant levels of lipid droplets in the SD vs. control mice (*p* < 0.001. Figure 1E). Taken together, these data suggested that SD increased susceptibility to AF and affected the atrial substrate.

### 3.2. Altered Gut Microbiota in SD Mice

**Figure 2:**
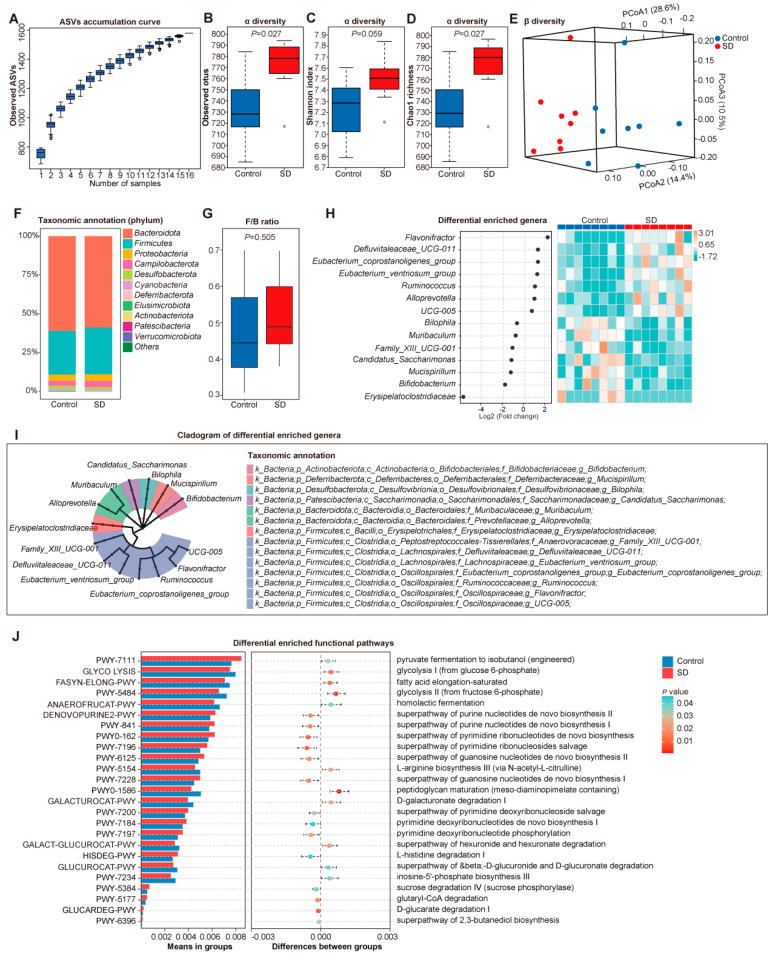
Altered gut microbiota in SD mice. (**A**) The ASVs accumulation curve. The observed OTUs (**B**), Shannon index (**C**), and Chao1 richness (**D**) in the control and SD group. Boxes represent the interquartile ranges; the inside line or points represent the median, and circles are outliers, Kruskal–Wallis test. (**E**) Principal coordinates analysis (PCoA) based on Bray–Curtis distance. The green circles represent control, and the purple circles denote SD. (**F**) Phylum-level taxonomic abundance and proportion for the control (left) and SD (right) groups; different taxa are differentiated by color. *n* = 8 for each group. (**G**) The ratio of the *Firmicutes* to *Bacteroidetes* (F/B ratio) in the control and SD groups. (**H**) Differential enriched genera between control and SD group, *t*-test. (**I**) The cladogram of differential enriched genera. (**J**) Differential enriched functional pathways between control and SD group, *t*-test.

The effects of SD on the fecal microbiota after 6 weeks of sleeping intervention were profiled by analyzing the DNA sequence encoding the 16S rRNA gene (*n* = 8 for controls, *n* = 8 for SD mice). The amplicon sequence variants (ASVs) accumulation curve was near saturation, which suggested that the sequencing data and the sample size were sufficient (Figure 2A). The observed operational taxonomic units (OTUs) (*p* = 0.027, Figure 2B), Shannon index (*p* = 0.059, Figure 2C), and Chao1 richness (*p* = 0.027, Figure 2D) were significantly higher in the SD group than in the control group, suggesting a significant shift in gut microbiota composition of SD. Moreover, the principal coordinates analysis (PCoA) based on Bray–Curtis distance among SD and control mice showed that microbiota community structures were significantly separated by SD at the first, second, and third principal coordinates (Figure 2E). In addition, considering the difference in body weight and blood pressure level between the two groups, we questioned whether the alterations of GM observed in the SD group were associated with these clinical factors. Thus, the independent strength of association between baseline factors and the GM signatures of alpha diversity was examined. The results showed that SD intervention was associated with increased Chao1 richness and observed OTUs independent of body weight or blood pressure level (Figure S1C). Therefore, it was concluded that the contribution of confounders to disordered GM was less than that of SD.

On the phylum level, *Bacteroidetes* decreased while *Firmicutes* increased along with SD (Figure 2F). Similar to a previous study that reported a significantly elevated ratio of the *Firmicutes* to *Bacteroidetes* (F/B ratio, an indicator of gut microbial dysbiosis) phyla in the gut of AF patients [14], an increased F/B ratio in SD mice was observed in this study (Figure 2G). These findings supported the premise that the increased susceptibility to AF might be associated with developing gut dysbiosis in SD.

On the genus level (Figure 2H,I and Table S1), the abundance of *Flavonifractor*, *Ruminococcus,* and *Alloprevotella* were higher, while the abundance of *Erysipelatoclostridiaceae*, *Bifidobacterium*, and *Mucispirillum* were lower in the SD group compared to the control group.

Furthermore, the functional potential of GM was predicted by PICRUSt (*t*-test, *p* < 0.05), and the results indicated that processes associated with pyruvate fermentation to isobutanol, glycolysis, and fatty acid elongation were over-represented in the bacteria of the SD group, while the function related to purine, pyrimidine and guanosine was over-represented in the control group (Figure 2J). In addition, the enzyme commission (EC) was analyzed, and 76 EC differed between SD and control (Table S2). These results suggest that the SD alters the gut microbial ecology of mice.

### 3.3. Changed Metabolic Patterns in Plasma of SD Mice

**Figure 3:**
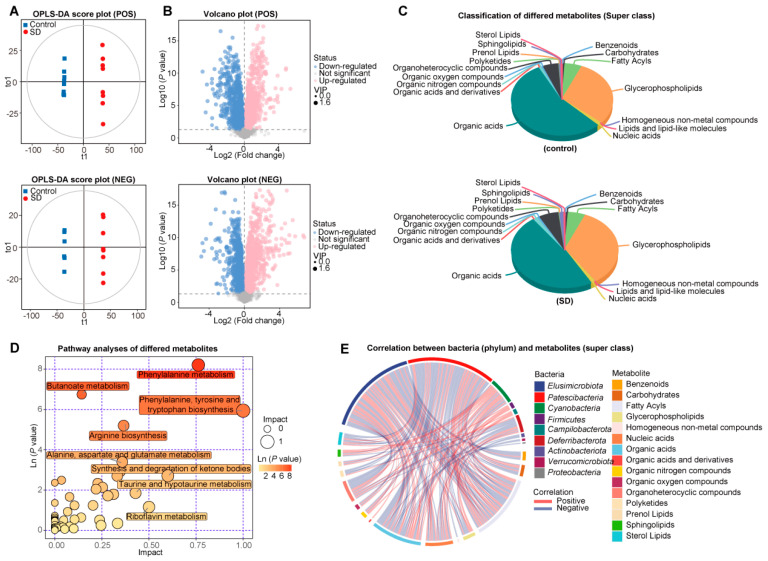
Changed metabolic patterns in plasma of SD mice. (**A**) Score scatter plots of orthogonal PLS-DA (OPLS-SA) comparing the plasma metabolic differences show the separation between control and SD in ES+ and ES−. ES+, positive ion mode; ES−, negative ion mode. (**B**) Volcano plots for the alterations of plasma metabolites between ES+ and ES− groups. Metabolite at *p* < 0.05 and variable importance (VIP) in the projection threshold of >1 was significantly different. Downregulation and upregulation were determined by fold-change. (**C**) Pie chart showing the proportion of metabolite annotated to the classification of superclass based on the metabolomics workbench. (**D**) Bubble analysis of metabolic KEGG pathways between control and SD group. (**E**) Circos plots showed Spearman’s correlation analysis (*p* < 0.05) between phyla and metabolites classified based on the superclass. Red indicates a positive correlation, and blue indicates a negative correlation. Each phylum and metabolic superclass were seen in a different color.

Metabolites provided the functional readout of cellular biochemistry; thus, the LC-MS-based metabolomic analysis was performed to clarify the effect of SD on plasma metabolite spectra of mice after 6-weeks of sleeping intervention (*n* = 8 for controls, *n* = 8 for SD mice). The orthogonal partial least squares–discriminant analysis (OPLS-DA) score plots showed a distinct separation in ES+ and ES− modes (Figure 3A). In addition, the permutation test showed no overfitting in the OPLS-DA model (Figure S2). Significantly differentially enriched metabolites were identified based on variable importance (VIP) in the projection threshold of >1 and *p* < 0.05 and were further matched in the Metlin database (Figure 3B). Overall, 102 plasma metabolites were enriched in SD, while 85 metabolites were increased in controls. According to the classification of superclass based on the metabolomics workbench, an increased portion of glycerophospholipids and decreased organic acids were found in the SD group (Figure 3C). 

Details of these metabolites are provided in Table S3. Kyoto Encyclopedia of Genes and Genomes (KEGG) metabolic pathway mapping (Figure 3D) showed that the altered metabolites were mainly involved in the primary metabolic pathways, such as phenylalanine metabolism and butanoate metabolism. In particular, various interesting trends were evident in the profiles of metabolites belonging to the same pathway (Table S4). For example, the levels of butanoate metabolism intermediates (i.e., 3-hydroxybutanoate, acetoacetic acid, 4-aminobutanoic acid, and butanoic acid) were decreased in SD mice.

Furthermore, straightforward associations between annotated phylums and metabolites based on superclass were demonstrated, indicating a complex linkage between bacteria and plasma metabolites (Figure 3E). Meanwhile, the correlation between differential enriched 76 bacterial EC and 187 metabolites was performed, and the results showed that 29 EC and 86 metabolites were significantly correlated with each other (FDR <0.01, |r| > 0.8). Thus, 86 metabolites may be significantly influenced by GM (Figure S3). These results suggest that SD intervention can alter the GM composition and plasma metabolites spectrum.

### 3.4. Modified Transcriptional Signatures in Atria of SD Mice

**Figure 4:**
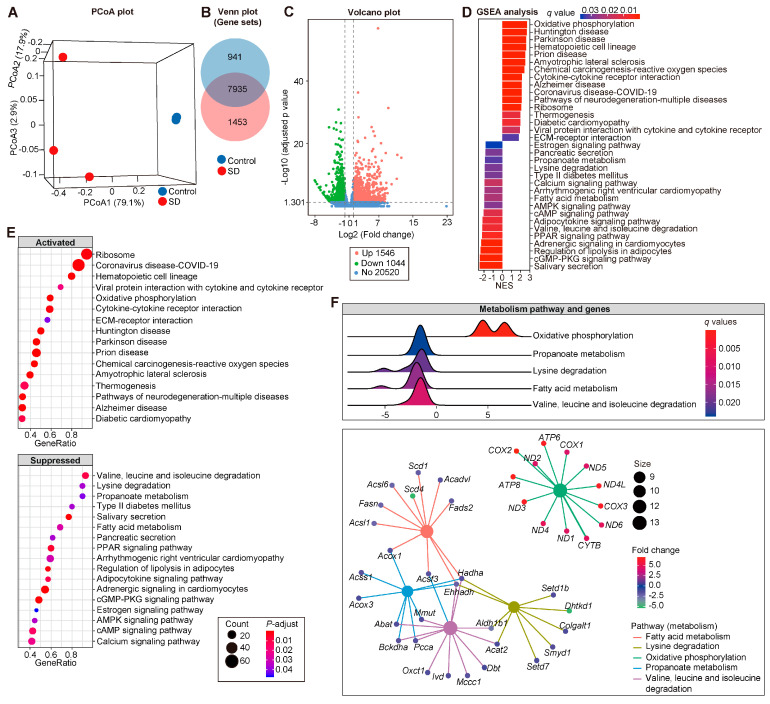
Modified transcriptional signatures in atria of SD mice. (**A**) Principal coordinates analysis (PCoA) based on Bray–Curtis distance. The green circles represent control and the purple circles denote SD. (**B**) Venn plot showed the mRNA detected in control and SD. (**C**) Volcano plots showing the results of DESeq2-based differential genes analyses. Adjusted *p*-value < 0.05 and |log2 (FoldChange)| > 1 were set as the threshold for significantly differential expression. Information of NES (**D**) and GeneRatio (**E**) of KEGG pathways revealed by the Gene Set Enrichment Analysis (GSEA) and the network (**F**) and ridge plot (**G**) of five metabolism-related pathways.

In parallel to metabolomic measurements, genome-wide analyses of changes in gene expression (RNA-sequencing) were performed. The atrium transcriptome of SD mice was different from the controls. A distinct separation was acquired from principal coordinate analysis (PCoA) (Figure 4A), which showed that SD remarkably impacts atrial transcriptomes. At the transcript level, 7935 mRNAs were detected in common between control and SD atrium (Figure 4B), among which 2590 mRNA were identified as the differential expressed genes (DEGs. Table S5), with 1546 upregulated and 1044 downregulated genes in SD compared with the control group (FDR < 0.05 and |log2FoldChange| > 1; Figure 4C).

Next, the GSEA analyses based on DEGs were performed to evaluate the responded functional pathway under the intervention of SD. Thirty-three KEGG pathways were annotated in SD (Figure 4D,E and Table S6); 16 pathways that were enriched, such as oxidative phosphorylation (NES = 2.7144), chemical carcinogenesis—reactive oxygen species (NES = 2.4081), and diabetic cardiomyopathy (NES = 1.9715), and 17 pathways were suppressed, such as propanoate metabolism (NES = −1.8942), fatty acid metabolism (NES = −1.9656), PPAR signaling pathway (NES = −2.2145), and regulation of lipolysis in adipocytes (NES = −2.3714). Hence, we concluded that SD influences the homeostasis of cardiac transcription regulation.

Considering the remarkable lipid accumulation in the atrium of SD, the pathways belonging to metabolism, including oxidative phosphorylation, propanoate metabolism, lysine degradation, fatty acid metabolism, and valine, leucine, and isoleucine degradation, as well as the 73 genes contained in the above-mentioned 5 pathways were extracted for further analysis (Figure 4F). Notably, some genes distributed at the crossing, such as *Acox1* (Peroxisomal acyl-coenzyme A oxidase 1), *Acsf3* (Acyl-CoA synthetase family member 3, mitochondrial), *Hadha* (Trifunctional enzyme subunit alpha, mitochondrial), *Ehhadh* (Peroxisomal bifunctional enzyme), *Mmut* (Methylmalonyl-CoA mutase, mitochondrial), *Abat* (4-aminobutyrate aminotransferase, mitochondrial), *Bckdha* (Branched chain ketoacid dehydrogenase e1, alpha polypeptide), *Pcca* (Propionyl-CoA carboxylase alpha chain, mitochondrial), *Aldh1b1* (Aldehyde dehydrogenase X, mitochondrial), and *Acat2* (Acetyl-CoA acetyltransferase, cytosolic) (Figure 4G), might bridge the complex linkage among SD-induced transcriptional responses in the atrium.

Meanwhile, the GSEA analyses about the Gene Ontology (GO) based on DEGs were carried out to explore the impact of SD on the atrial biological process (BP). Overall, 222 GO-BP were acquired (Table S7), and 12 GO-BPs with q < 0.01 and absolute NES ≥ 3 are shown in Figure S4A,B, including GO:0044057, regulation of system process; GO:0003015, heart process; GO:1903522, regulation of blood circulation; GO:0060047, heart contraction; GO:0008016, regulation of heart contraction; GO:0006941, striated muscle contraction; GO:0006942, regulation of striated muscle contraction; GO:0060048, cardiac muscle contraction; GO:0086001, cardiac muscle cell action potential; GO:0086003, cardiac muscle cell contraction; GO:0055117, regulation of cardiac muscle contraction; and GO:0086065, cell communication involved in cardiac conduction. 

In addition, genes such as *Ank2*, *Ctnna3*, *Dsc2*, *Dsg2*, *Dsp*, *Gja5*, *Hcn4*, *Ryr2*, *Scn5a*, and *Kcnq1*, were annotated in multiple GO-BPs (Figure S4C), and most of them were associated with the electrical homeostasis, which may have a crucial role in the formation of an arrhythmogenic substrate.

### 3.5. The Linkage between GM, Plasma Metabolism, and Atrial Transcriptome

To explore the potential linkage between plasma metabolites and atrial genes, the Spearman correlation analysis between 73 DEGs originating from 5 metabolism pathways belonged to KEGG pathways and 86 metabolites filtered after significant correlation with GM-related EC (FDR < 0.01, |r| > 0.8) was performed (Table S8). Overall, 67 genes and 84 metabolites were significantly correlated with each other. Based on the standard of degrees, more than three, 65 genes (Table S9) and 74 metabolites were retained for further analyses (Figure S5). Next, a string was used to explore the protein–protein interaction networks of 65 overlapping genes. Thirty-six pathways (Table S10), such as propanoate metabolism, fatty acid metabolism, etc., were enriched in total (Figure S6).

## 4. Discussion

The present study demonstrated that SD exposure for 6 weeks increases AF susceptibility in mice. It also aggravates fibrosis, collagen, and lipid accumulation in the atrium, alters GM, leads to microbial function imbalance, alters various primary metabolomic pathways in plasma, and disrupts metabolic homeostasis in the atrium. These results demonstrated that the crosstalk between GM and atrial metabolism might be the potential promising target for SD-mediated AF susceptibility.

AF, as the most common arrhythmia in clinical practice, has been associated with increased morbidity, mortality, and healthcare burden [33]. Recent studies suggested the importance of sleep disturbance in regulating the process of risk factors for AF development, including hypertension, heart failure, diabetes, and obesity [4,5,6,33], all of which correspond to GM dysbiosis [34]. Meanwhile, the direct interaction between disordered GM and AF has been confirmed [17]. The dysbiosis of GM composition and function is closely linked to AF onset, duration, type, and recurrence [14,15,16,35], especially the imbalance of GM-derived metabolites, including lipopolysaccharide (LPS), trimethylamine N-oxide (TMAO), SCFAs, and bile acids (BAs), which are involved in inflammation, fibrosis and disordered lipid metabolism, and may lead to AF progression [11,17,36,37,38,39]. A recent large-scale 10-year follow-up cohort study [7] suggested that longer lifetime durations and 3–8 nights/month frequency of night shift work exposure are associated with higher AF risk. However, the potential mechanism of sleep disturbance increasing incident AF risk and the influence of GM remain unclear. The results from recent human cohorts indicate that the gut microbiota–bile acid axis may link the positive association between chronic insomnia and cardiac disorders. *Ruminococcaceae UCG-002* and *Ruminococcaceae UCG-003* are the main genera mediating the positive association between chronic insomnia and cardiometabolic diseases [40]. Notably, the enrichment of *Ruminococcus* was identified in the current SD mice, and the potential mechanism mediating the impact of gut microbes on the atrial substrate should be further investigated.

Other studies found that disrupted adaptability to sympathetic nerve excitement and excessive norepinephrine secretion during sleep restriction enhance myocardial contractility and increase conduction speed and oxygen consumption, thus further aggravating the load on the heart [41,42]. Furthermore, increased cardiovascular, inflammatory markers such as interleukin-6, C-reactive protein, and tumor necrosis factor α [43,44], as well as the degree of oxidative stress damage [45], which are all linked to atrial remodeling [46], have been reported during sleep restriction.

In this study, we further examined the effect of SD on atrial histological changes, gut flora composition, plasma metabolic spectrum, as well atrial transcriptomic deciphered in a mouse model. The association between chronotype and GM has been recently recognized. Triplett et al. examined temporal and region-specific effects of sleep fragmentation on GM and intestinal morphology in Sprague Dawley rats, finding that both acute (6 days) and chronic (6 weeks) sleep fragmentation (SF) in rats, induces GM dysbiosis, accompanied by increased crypt depth in the distal ileum and an increase in the number of villi lining. In addition, chronic SF induces decreased microbial adhesion and penetration in the distal ileum and cecum [47]. Emerging evidence suggests that sleep disturbance braked multiple homeostases mediated by GM. Transplantation of the “sleep deprivation microbiota” into germ-free mice activated the Toll-like receptor 4/nuclear factor-κB signaling pathway, peripheral and central inflammatory processes, and impaired cognitive function in the recipient mice [48].

Besides the direct effect of GM-mediated sleep disturbance-induced disease progression, newly emerging data demonstrate that microbiota has circadian rhythmicity, which is tightly interrelated to the host circadian rhythm [49]. Altered microbiota rhythmicity has been associated with the host circadian clock gene network [50,51]. Meanwhile, food is a dominant cue for bacterial rhythms, independent of the clock gene network [52,53]. For example, GM-derived TMAO can influence the expression of circadian clock genes in endothelial cells [54]. Thus, a dynamic interaction exists between the host circadian system and the microbiota. Disruptions in this relationship have important consequences for host metabolism and could be one mechanism through which the microbiota is involved in metabolic syndrome and related pathologies. Furthermore, a recent study suggested that cohort-specific risk pattern of arrhythmic taxa enables the classification and can predict the risk of type 2 diabetes mellitus, suggesting a functional link between circadian rhythms and the microbiome in metabolic diseases [55].

SCFA was detected in the current multi-omics analyses in the aspects of metabolic pathways such as butanoate metabolism, propanoate metabolism, as well as acetate-included pyruvate metabolism, and glycolysis /gluconeogenesis. SCFA, the metabolite derived from gut microbial fermentation, is an important factor in the microbiota–gut–brain axis and circadian rhythms, acting on multiple mechanisms, including modulating the host metabolism [56]. Specifically, SCFA has been reported to be associated with increased oxygen consumption and fat oxidation [57], as well as reduced lipid synthesis and lipid accumulation in the liver and adipose tissue [58]. SCFA signaling is a credible mechanism connecting the microbiota to metabolic dysfunction and metabolic syndrome. Another research reported that sleep apnea-induced arterial blood pressure elevation and aortic and coronary artery function impairments could be mitigated by probiotic administration [59].

Notably, lipid accumulation in the atrium was identified in the current study. The heart is an energy-demanding organ relying on fatty acid (FA) and glucose oxidation. A failing heart usually shows impaired transcription of key enzymes involved in FA metabolism and impaired mitochondrial oxidative function [60,61,62]. Consequently, the heart switches from FA to utilizing glucose as the main fuel. Interestingly, the current multi-omic data revealed that several genes, such as *Ehhadh*, *Acox3*, and *Acox1*, were found to be at the crossroads of multiple pathways which might act as a coordinator. For example, *Ehhadh*, Enoyl-CoA, hydratase/3-hydroxyacyl CoA dehydrogenase, regulated and mediated by PPARα, encodes a bifunctional enzyme protein essential for the peroxisomal β-oxidation pathway for the breakdown of very long chain fatty acids and indispensable for the production of medium-chain dicarboxylic acids [63]. Thus, the decreased *Ehhadh* in the SD group’s atrium might be associated with altered fatty acid metabolism and pathologic remodeling [64]. Furthermore, *Acox3* (acyl-Coenzyme A oxidase 3) and *Acox1* (acyl-Coenzyme A oxidase 1) are essential genes related to β-oxidation; therefore, the decreased profiling promotes the disturbed metabolism of the myocardium, which is consistent with the findings from diabetic cardiac microvascular endothelial cells [65].

The present study still has some limitations. First, the current study confirms correlation but not causation. Thus, further studies with fecal transplantation might provide a stronger conclusion explaining AF-induced GM dysbiosis and the progression of AF. Furthermore, quantifying plasma and myocardial triglycerides and free fatty acids levels, as well as myocardial lipidomic, might provide more information about altered lipid profiles in the SD atrium. Moreover, further experiments on optical and electrical mapping, atrial refractoriness, and patch clamp-based ion channel studies are needed to assess the impact of SD on the electrophysiological features of the atrium and atrial myocardial cells. In addition, further studies are required to explore whether supplementation of SCFA to SD mice could alleviate the concomitant atrial remodeling and protect from AF. 

To sum up, our data suggest that SD leads to enhanced AF inducibility and atrial injury, especially lipid accumulation, accompanied by alterations in GM structure, circulating metabolomics, and atrial transcriptomic patterns. These results provide preliminary evidence that the crosstalk between GM and atrial metabolism might be the potential promising target for sleep disturbance-mediated AF susceptibility. Yet, more research is needed to further confirm these findings.

## Data Availability

The source data for 16s rRNA sequencing has been deposited in the Sequence Read Archive (SRA) under the BioProject accession code PRJNA859441 and other datasets analyzed in this study are available from the corresponding author on reasonable request.

## Supplementary Materials

The following supporting information can be downloaded at: https://www.mdpi.com/article/10.3390/metabo12111144/s1, Figure S1: Body weight and blood pressure of the experimental mice; Figure S2: The permutation test of the OPLS-DA model; Figure S3: Correlation heatmap between metabolites and ECs; Figure S4: GSEA analysis about GO-BP based on differential expressed genes; Figure S5: Correlation heatmap between 65 DEGs and 74 metabolites; Figure S6: Protein–protein interaction network for 65 DEGs extracted from 5 metabolism pathways; Table S1: Relative abundance of annotated genera; Table S2: Relative abundance of ECs; Table S3: Relative abundance of differed metabolites; Table S4: Pathway analyses of differed metabolites; Table S5: Information of differential expressed genes; Table S6: GSEA analysis about KEGG pathways based on differential expressed genes; Table S7: GSEA analysis about GO-BP based on differential expressed genes; Table S8: Correlation analyses between 73 DEGs and 86 metabolites; Table S9: Sixty-five overlapping genes acquired from metabolomics and transcriptomics data; Table S10: Mapped KEGG pathways by String.
