# Supplementary figures and images for: The Impact of Sleep Disturbance on Gut Microbiota, Atrial Substrate, and Atrial Fibrillation Inducibility in Mice: A Multi-Omics Analysis
Source: Metabolites. 2022 Nov 20;12(11):1144. doi: 10.3390/metabo12111144 (PMC9694206; doi:10.3390/metabo12111144)

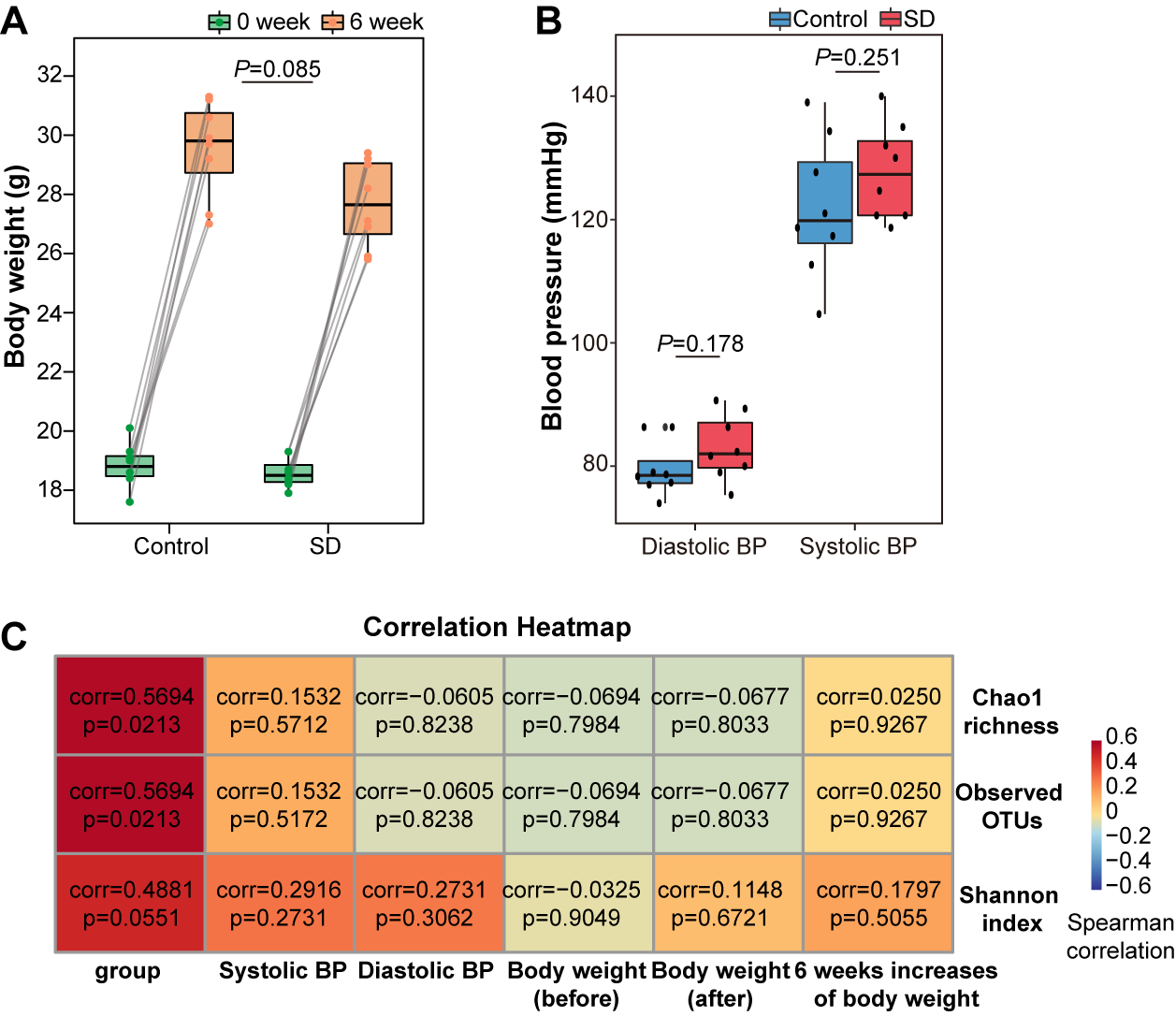

Supplement: Supplementary file 1 [file metabolites-12-01144-s001.zip › Figure S1.tif]

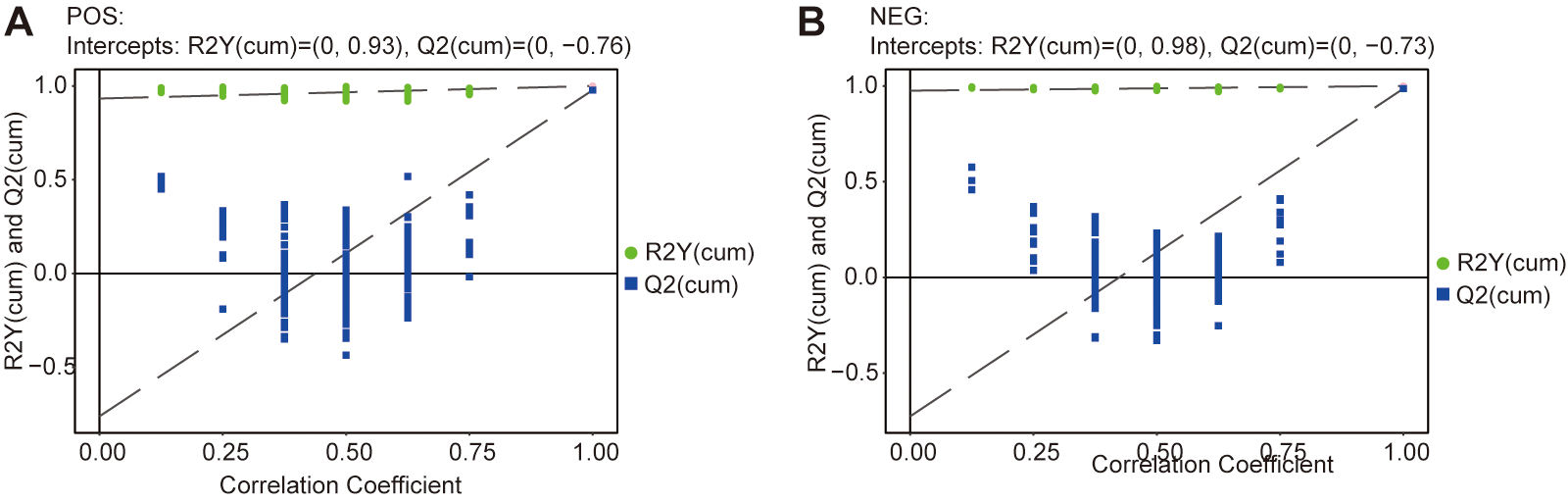

Supplement: Supplementary file 1 [file metabolites-12-01144-s001.zip › Figure S2.tif]

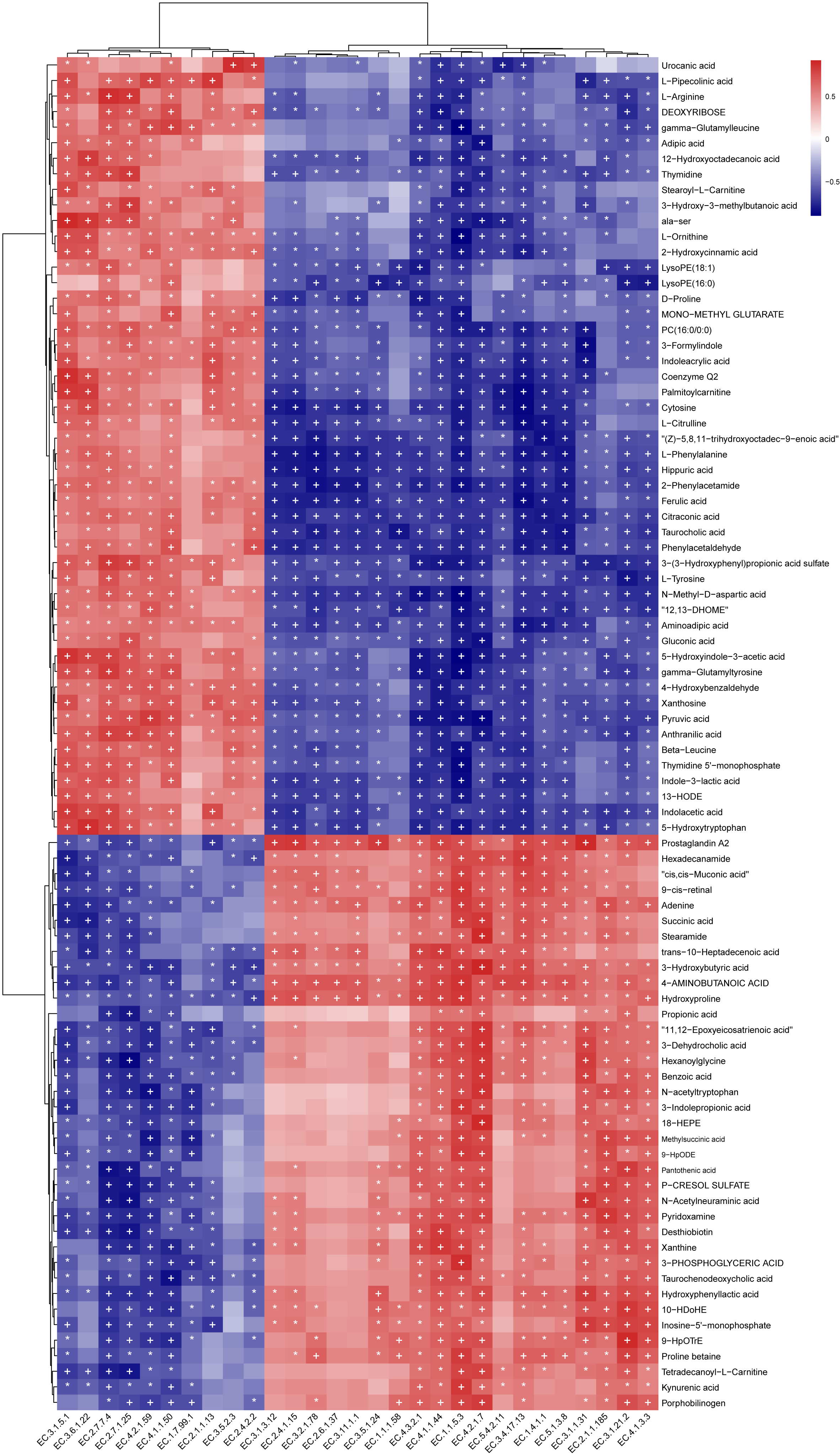

Supplement: Supplementary file 1 [file metabolites-12-01144-s001.zip › Figure S3.tif]

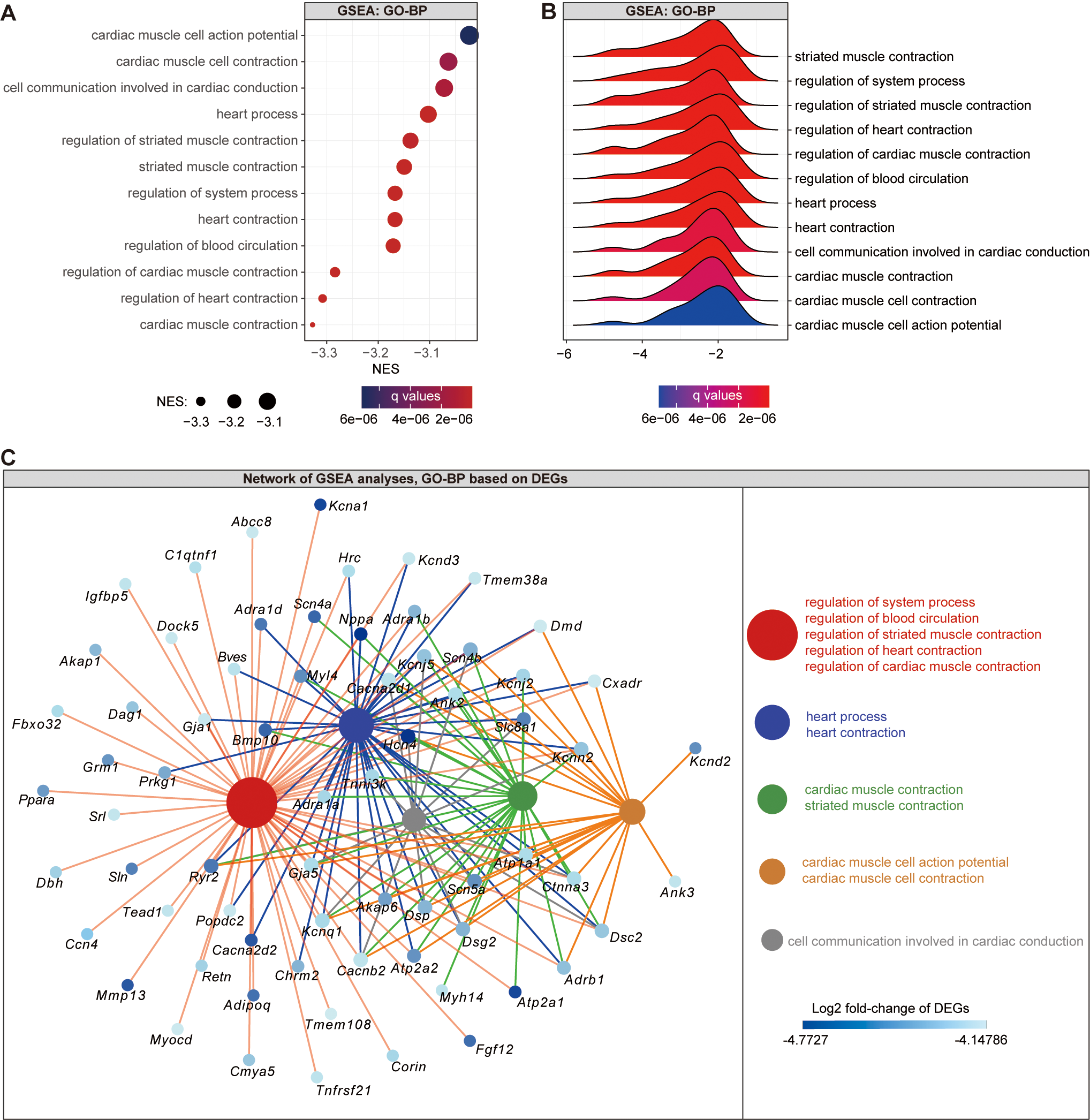

Supplement: Supplementary file 1 [file metabolites-12-01144-s001.zip › Figure S4.tif]

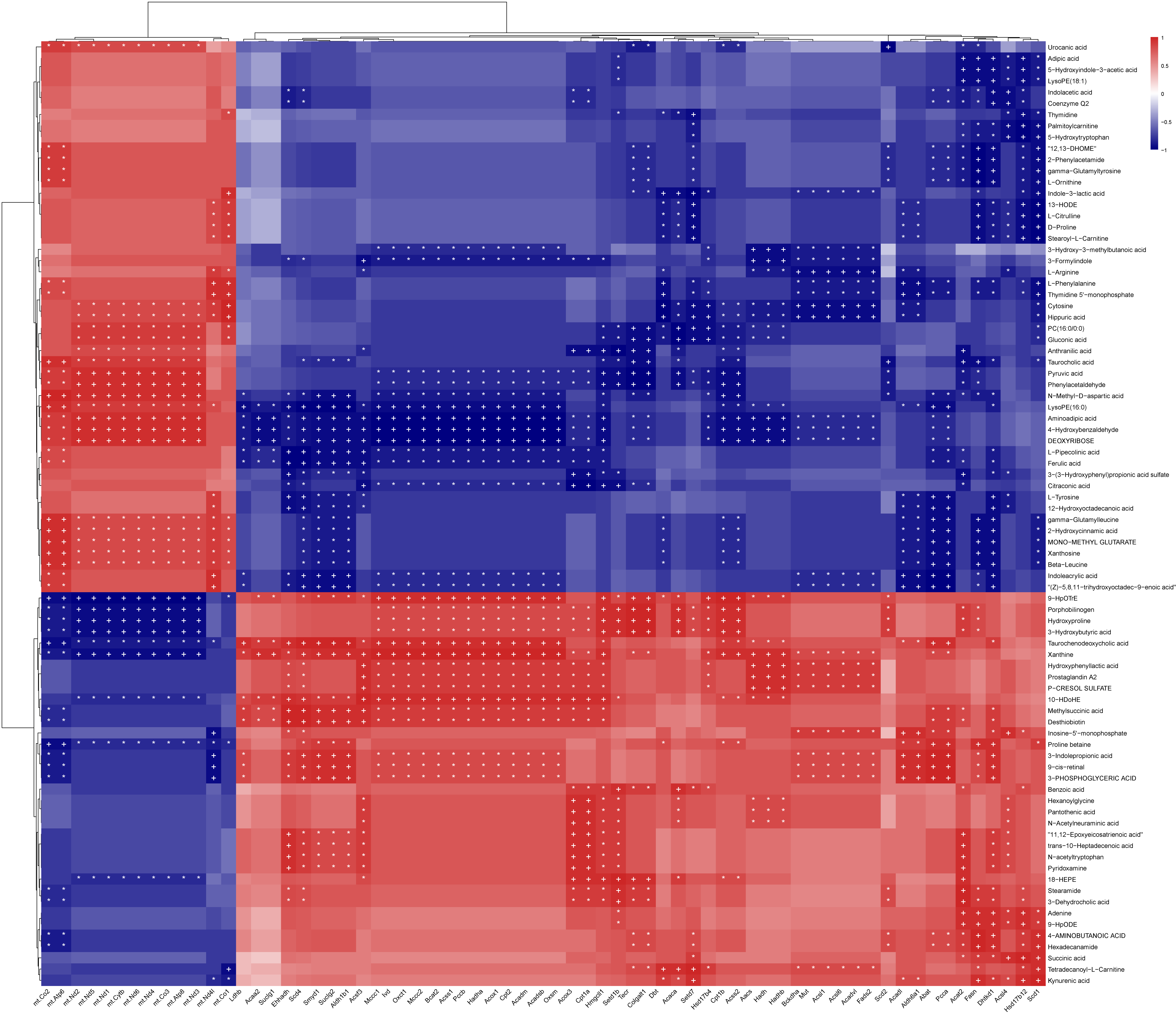

Supplement: Supplementary file 1 [file metabolites-12-01144-s001.zip › Figure S5.tif]

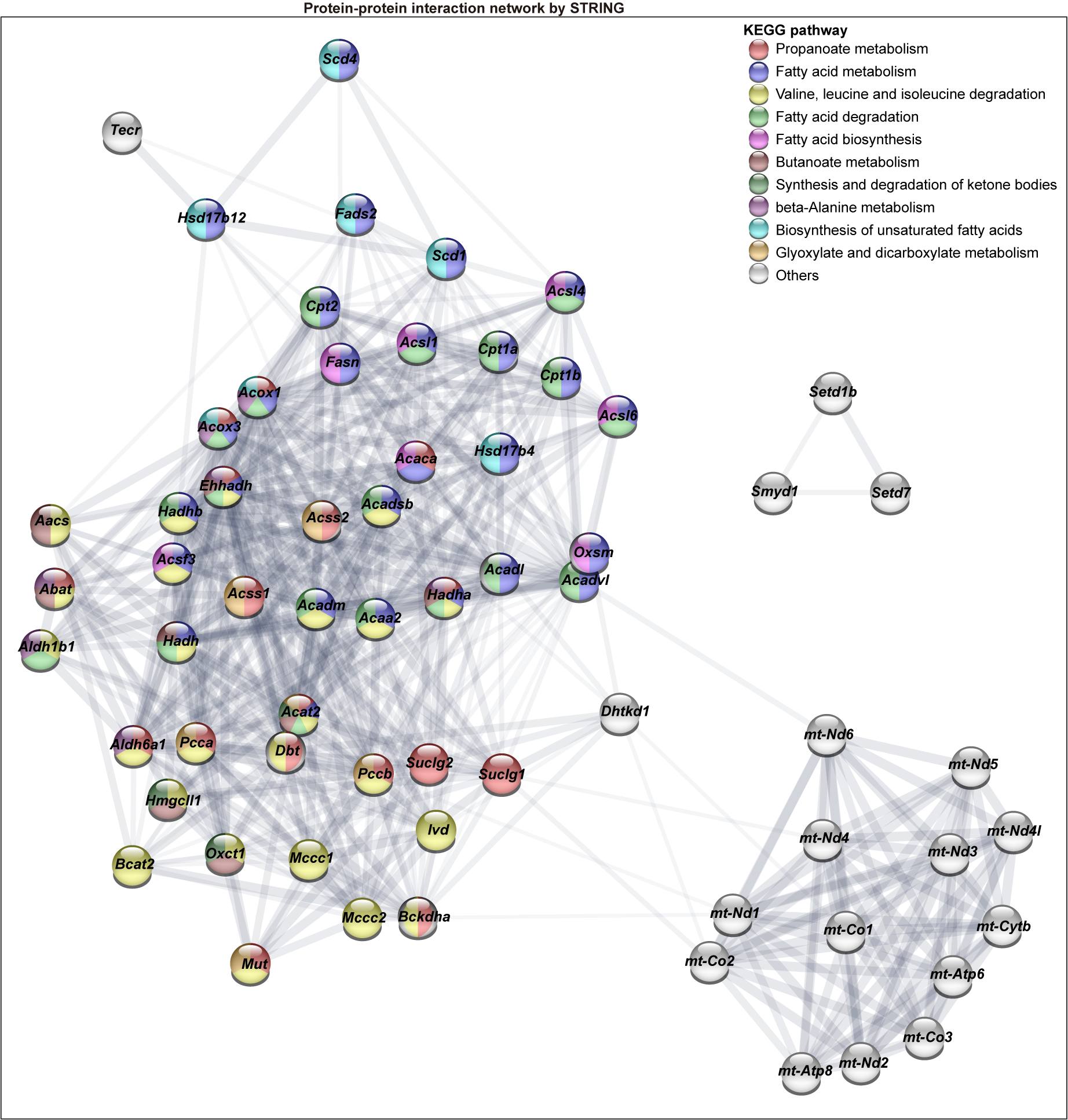

Supplement: Supplementary file 1 [file metabolites-12-01144-s001.zip › Figure S6.tif]
